# Sea Sickness

**Published:** 1868-11

**Authors:** 


### SEA SICKNESS.

We have, in back numbers of the Journal, repeatedly given advice in regard to encountering and resisting the effects of this disagreeable malady, based on our own experience. That the sufferings of some people when sea sick are fearful, we have had ample occasion to observe. We made one voyage to Europe—landing in one of the Mediterranean ports—in company with an unfortunate lady who, voyaging in the hope of benefit to her lungs, was horribly sea sick; before she reached Sandy Hook she took to her berth, and was out of it but two or three times during the whole passage, which occupied sixty days. Twice during severe spasms of retching we supposed that her spirit had fled, but the application of a mustard poultice over what is called the pit of the stomach, restored her to consciousness again.

We know, therefore, that many persons in delicate health would be spared an immense amount of suffering, if they could make a sea voyage without being sea sick, and for the benefit of such, we propose to speak here of the new discovery, by Dr. Chapman of England, of the method of applying *bags filled with ice* to the spine to prevent sea sickness.

Dr. Chapman claims for his discovery, that

"As a general rule, liable to few exceptions, the effect of this simple expedient will be the annihilation of all unpleasant symptoms; the sickness will stop; if diarrhœa is present, it will be subdued: if the patient is only threatened with it, the attack will be averted; if there be headache, with coldness of the forehead, the pain will vanish; the cold clammy sweat will cease to exude; the cold skin will become warm again, the muscular system will regain its usual strength; the mind will recover its energy and pleasurable interest in surrounding objects; and the sickly, pallid features will resume their expressive energy and healthy hue."

If these results can be obtained, the discovery will prove a great boon to many a poor creature who is obliged "to go down to the sea in ships."

We subjoin Dr. Chapman's rules for the preparation and use of these ice-bags, only, adding that while the remedy is vouched for by respectable physicians in England and America, we know nothing of it from personal observation.

FIRST.—The bag into which the ice is placed is made of india-rubber, and for convenience' sake is divided into three compartments by india-rubber septa, its mouth being closed by a brass clamp.

SECOND.—The three divisions are respectively filled with small pieces of ice about the size of a walnut, care being taken that it is not packed too closely, or it will not touch the back in its whole extent.

THIRD.—The bag is best applied by lying on it.

FOURTH.—In cases of sea sickness, it is to be applied from the top of the neck to the lower part of the hollow of the back. It will generally be sufficient if it is placed on the outside of the sheets or chemise, but it will be more certain in its action, if placed in direct contact with the skin. It may be readily pushed into position without much derangement of the dress.

FIFTH.—The part on which it is most important to keep it closely applied is that portion of the spine, extending from the lower angle of the shoulder-blades to the lower part of the back. Great care should be taken to prevent the bag slipping up to the back of the head. If headache be produced, and if the forehead be at the same time distinctly warmer than usual, a folded handkerchief should be placed between the upper part of the bag and the skin.

SIXTH.—Persons naturally liable to sea sickness should not only apply the bag directly to the skin, but also for half an hour or an hour before they go on board.

SEVENTH.—The ice should be applied uninterruptedly, without even the intermission of a few minutes, and for this purpose, it is advisable on long voyages to be supplied with more than one bag to prevent delay in changing.

EIGHTH.—Persons with delicate chests and subject to coughs should use the remedy with caution and under medical advice, as Dr. Chapman believes that it is likely to produce congestion of the lungs and increase of cough in people with phthisical tendencies, if applied along the dorsal vertebra *when they are not sea sick.*

We apprehend that the necessity for Rule VIII shows that whatever may be said in favor of the remedy, it must be used with the greatest caution. Remember, reader, that we do not recommend it; but deem it to be our duty to record the discovery. The same application has also been found to be almost a specific in Asiatic Cholera.

